# Is there a difference between presence of single stone and multiple stones in flexible ureterorenoscopy and laser lithotripsy for renal stone burden <300mm^2^?

**DOI:** 10.1590/S1677-5538.IBJU.2015.0646

**Published:** 2016

**Authors:** Faruk Ozgor, Onur Kucuktopcu, Burak Ucpinar, Zafer Gokhan Gurbuz, Omer Sarilar, Ahmet Yalcin Berberoglu, Murat Baykal, Murat Binbay

**Affiliations:** 1 Department of Urology, Haseki Training and Research Hospital, Istanbul, Turkey

**Keywords:** Catheters, Kidney Calculi, Urolithiasis

## Abstract

In this study, we aim to evaluate and compare the effectiveness of flexible ureterorenoscopy (f-URS) for solitary and multiple renal stones with <300 mm^2^ stone burden. Patients' charts who treated with f-URS for kidney stone between January 2010 and June 2015 were reviewed, retrospectively. Patients with solitary kidney stones (n:111) were enrolled in group 1. We selected 111 patients with multiple kidney stones to serve as the control group and the patients were matched at a 1:1 ratio with respect to the patient's age, gender, body mass index and stone burden. Additionally, patients with multiple stones were divided into two groups according to the presence or abscence of lower pole stones. Stone free status was accepted as complete stone clearence and presence of residual fragments < 2 mm. According to the study design; age, stone burden, body mass index were comparable between groups. The mean operation time was longer in group 2 (p= 0.229). However, the mean fluoroscopy screening time in group 1 and in group 2 was 2.1±1.7 and 2.6±1.5 min, respectively and significantly longer in patients with multiple renal stones (P=0.043). The stone-free status was significantly higher in patients with solitary renal stones after a single session procedure (p=0.02). After third month follow up, overall success rate was 92.7% in Group 1 and 86.4% in Group 2. Our study revealed that F-URS achieved better stone free status in solitary renal stones <300 mm^2^. However, outcomes of F-URS were acceptable in patients with multiple stones.

## INTRODUCTION

Recently, Shock Wave Lithotripsy (SWL) is recommended as one of the first line treatment modalities for renal stones under 300mm^2^ stone burden, according to European Urology Association Guidelines (1). Although, SWL has high success rates and higher patient compliance, effectiveness of SWL tends to decrease in lower pole stone(s), hard stone(s) such as calcium oxalate monohydrate stones and multiple stones (2). On the other hand, percutaneous nephrolithotomy (PNL) has favourable results for renal stone(s) regardless of stone type, but procedure has potential serious complications including bleeding necessitating transfusion, septicaemia and colonic injury (3). Additionally, multiple stones may require multiple accesses which may increase the number of complications.

With advancements in endoscopic technology and increasing surgical experience, flexible ureterorenoscopy (f-URS) has become an important treatment modality for renal stones(s). Modern flexible ureterorenoscopes may access all pelvicaliecal system including lower pole, and Holmium Laser provides effective stone fragmentation regardless of stone type. Additionally, use of ureteral access sheath facilitate the passage of stone fragments, which improves patients quality of life after the procedure (4). Many authors had achieved up to 90% stone free rates with acceptable complications in f-URS procedure in moderate sized stones (5, 6).

Although there are many studies showing the effectiveness of f-URS for solitary renal stones, studies evaluating the effectiveness of f-URS in multiple renal stones is limited. In this study, we aimed to evaluate and compare the efficacy and safety of f-URS for solitary and multiple renal stones with <300mm^2^ stone burden. To our knowledge, this is the first study comparing the effectiveness of f-URS for solitary and multiple renal stones.

## MATERIALS AND METHODS

In a single tertiary academic center, 413 patient's charts who were treated with f-URS for kidney stone between January 2010 and June 2015 were reviewed, retrospectively. However, patients datas were recorded prospectively. Patients who had kidney stone burden <300mm^2^ were enrolled into the study. Stone burden was calculated according to European Association of Urology guideline's formula (1). Patients with a solitary kidney stone and multiple kidney stones were divided as group 1 and group 2, respectively. At the end of our evaluation, 111 patients enrolled in group 1. We selected 111 patients with multiple kidney stones to serve as the control group and the patients were matched at a 1:1 ratio with respect to the patient's age, gender, presence of solitary kidney and stone burden. Patients under 18 years of age and patients with renal abnormalities were excluded from the study. Additionally, to evaluate the effect of lower pole stone on f-URS outcomes, patients with multiple stones were divided into two groups according to the presence or absence of lower pole stones.

In all patients, detailed medical history was obtained and physical examination was performed. Preoperatively, renal stone and kidney characteristics were assessed by intravenous urography (IVU) and/or computed tomography (CT). Patients demographic parameters including sex, age, body mass index (BMI), degree of hydronephrosis, stone size, stone number and location were recorded. Hemoglobin measurements, serum creatinine level, platelet counts and coagulation screen tests were assessed preoperatively. All patients had sterile urine culture prior to procedure. Finally, all patients signed an informed consent form before surgery.

### f-URS technique

In all cases, a standardized f-URS procedure was performed by well-trained surgeons. After induction of general anesthesia, semirigid ureteroscopy was performed for visual assessment of ureter and facilitate the placing of ureteral access sheath. 8.7F digital flexible ureteroscope (DUR-D Gyrus ACMI, Southborough, MA, USA) or a 7.5F fiber-optic (Storz FLEX-X 2, Tuttlingen, Germany) with a 200 or 273µm laser fiber were used for treatment. Stone fragmentation was performed with holmium laser with an energy of 0.8 – 1.5J and a rate of 5 – 10Hz. Stone fragments <2mm were left for spontaneous passage and basket retrieval was performed for >2mm stone fragments. At the end of each procedure, semi-rigid ureteroscopy was performed to check the integrity of the ureter and a 4.8F double-J catheter was routinely placed in each patient. Operation time was accepted as the time passed from insertion of the flexible ureterorenoscope to the completion of double-J catheter placement. Our patients were discharged from the hospital on first postoperative day. In the 3^rd^ week of operation, double-J catheter was removed. Additional procedures were recommended to our patients if residual stone fragments bigger than 4mm were identified.

Initial postoperative stone-free status was evaluated at hospital discharge with a kidney – ureter – bladder radiogram. In follow-up, stone-free rates were determined in an outpatient clinic setting at 3 months postoperatively with low-dose spiral CT. Stone free status was accepted as complete stone clearence and presence of residual fragments less than 2mm.

During statistical analyses values were evaluated as numbers, means, percentages and intervals. Numbers and percentages were compared using Chi-square test. Before the comparison of means of values, the values were evaluated for homogenity. Homogenously distributed values were compared using Student T test and heterogenously distributed values were compared using Mann Whitney U test.

## RESULTS

According to the study design, gender, age, BMI and stone burden were similar between two groups (p=1, p=0.924, p=0.592, p=0.936, respectively). Additionally, degree of hydronephrosis, stone opacity and presence of solitary kidney were comparable between groups (p=0.363, p0.065=, p=0.531, respectively). The mean stone number was 2.38 in Group 2. Preoperative parameters are summarized in Table-1.

**Table 1:** Pre-Operative Characteristics.

		Single stone	Multiple stones	P value
Number		111	111	
Mean age (years)		46.1±14.1	45.9±13.9	0.924
Mean body mass index (kg/m^2^)		26.6±4.5	26.3±5.2	0.592
Mean stone area(mm^2^)		155.3±45.2	155.9±60.2	0.936
Gender				1
	Male	55.9%	55.9%	
	Female	44.1%	44.1%	
Operation side				0.351
	Left	51.5%	53.2%	
	Right	49.5%	46.8%	
The mean stone number				<0.001
		1	2,38	
Hydronephrosis				0.363
	0	37	28	
	1	43	41	
	2	22	35	
	3	7	6	
	4	1	1	
Solitary Kidney		6.4%	4.5%	0.531
Stone opacity				0.065
	Opaque	100	106	
	Non opaque	11	5	
Previous SWL history		45%	53.2%	0.314

**SWL** = Shock Wave Lithotripsy

The percentage of different scope type usage (fiberoptic or digital) were similar between groups (p=0.299). Although the mean operation time was longer in group 2, there was no statistically significant difference (p=0.229). However, the mean fluoroscopy screening time in group 1 and in group 2 was 2.1±1.7 and 2.6±1.5 min, respectively and significantly longer in patients with multiple renal stones (P=0.043). Stone relocation from lower pole to the pelvis or to another appropriate calyx was performed in 68.8% of our patients. The mean hospitalization time of our patients were 20.6±14.5 and 22.5±16.8 in group 1 and group 2, respectively.

**Figure 1:**
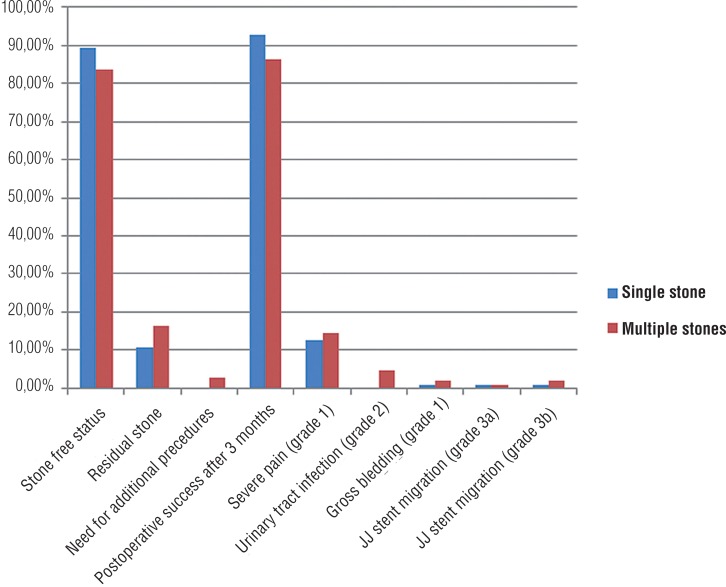
Comparison of post operative stone free status and complications between patients with single stone and multiple stones.

The complications were graded according to the Clavien-Dindo classification (7). Although complication rates were slightly higher in group 2, the difference was not statistically significant. Post operative severe pain was the most common unfortunate event that was seen in 14 (12.6%) and 16 (14.4%) patients in group 1 and group 2, respectively. Post-operative fever requiring the change of antibiotic therapy was seen in 5 patients of group 2. Severe bleeding that resolved spontaneously in follow-up occured in one patient in group 1 and in two patients of group 2. None of our patients required blood transfusion. Double-J catheter migration occurred in five patients (2 patients in group 1 and 3 patients in group 2). The double-J catheter was inserted under local anesthesia in two patients and under general anesthesia in three patients, respectively (Figure-1).

The stone-free status was significantly higher in patients with solitary renal stones when compared to patients with multiple renal stones (89.2% vs. 83.8%) after a single procedure (p=0.02). A second f-URS was required for 3 patients in group 2. These patients were completely stone free. After three months of follow-up, overall success rate was 92.7% in group 1 and 86.4% in group 2 (Table-2). Additionally, stone compositions are listed in Table-2.

**Table 2:** Operative and post-operative characteristics.

	Single stone	Multiple stones	P value
Number	111	111	
Type of f-URS			0.299
Flex-X2 (Fiberoptic)	94	87	
Dur-D (Digital)	17	24	
Mean operation time(minutes)	44.6±16.8	47.8±22.2	0.229
Mean fluoroscopy time(minutes)	2.1±1.7	2.6±1.5	0.043
Mean hospitalisation time(hours)	20.6±14.5	22.5±16.8	0.387
Post operative complications			0.282
Severe pain (grade 1)	14 (12.6%)	16 (14.4%)	
Need for antibiotic change due to fever (grade 2)	0%	5(4.5%)	
Gross bleeding (grade 1)	1(0.9%)	2(1.8%)	
JJ Catheter migration (grade 3a)	1(0.9%)	1 (0.9%)	
JJ Catheter migration (grade 3b)	1 (0.9%)	2 (1.8%)	
Postoperative success after single session			0.020
Stone free status	99 (89.2%)	93(83.8%)	
Residual stone	12(10.8%)	18(16.2%)	
Need for additional procedures	0%	3(2.70%)	0.385
Postoperative success after 3 months	103 (92.7%)	97 (86.4%)	0.016
Stone analysis			0.865
Calcium oxalate monohydrate	28	37	
Calcium oxalate dihydrate	11	15	
Uric acid	4	2	
Cystine	4	7	
Struvite	1	2	
Mixt	8	13	

**JJ catheter** = Double-J catheter

**Figure 2:**
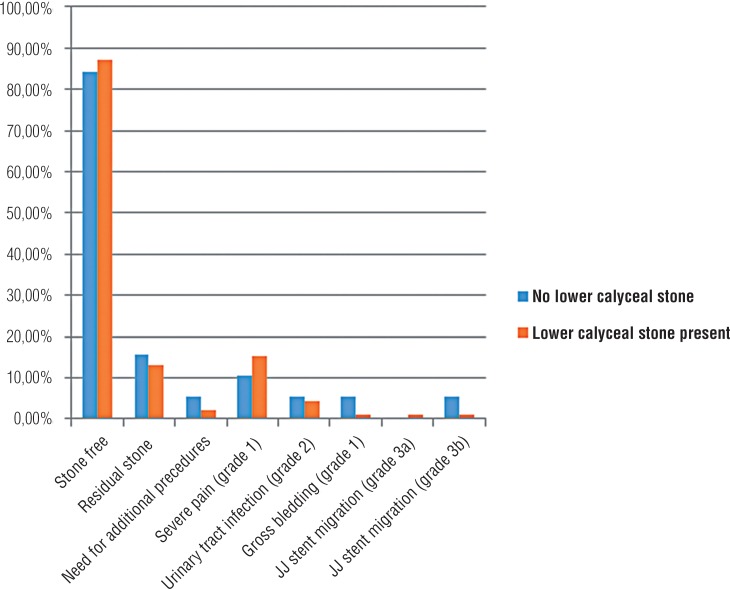
Comparison of post operative stone free status and complications between patients with and without lower calyceal stone, in multiple stone group.

In group 2, 92 patients with lower pole stone and 19 patients without lower pole stone were categorized in group 2a and in group 2b, respectively. Demographic characteristics were well-matched between groups (Table-3). Also, mean operation time and mean fluoroscopy screening time was similar (p=0.431 and p=0.436, respectively). Complication rates were not significantly different between groups (p=0.616). Stone free status was 87.0% in group 2a and 84.2% in group 2b after a single procedure (p=0.856) Figure-2). Second look f-URS was performed in two patients and one patient in group 2a and in group 2b, respectively (Table-4).

**Table 3:** Pre-Operative characteristics of patients with multiple renal stones according to absence/presence of lower pole stone.

		Lower calyceal stone present	No lower calyceal stone	P value
Number		92	19	
Mean age (years)		45.8±13.9	46.6±14	0.803
Mean body mass index (kg/m^2^)		26.7±5.5	24.3±2.4	0.075
Mean stone area(mm^2^)		156.4±60	153.3±63.1	0.839
Gender				0.086
	Male	26.3%	73.7%	
	Female	47.8%	52.2%	
Operation side				0.211
	Left	54.3%	31.6%	
	Right	45.7%	68.4%	
Stone localisation				<0.001
	Multiple stones with lower pole stone	92	0	
	Multiple stones without lower pole stone	0	19	
Hydronephrosis				0.218
	0	23	5	
	1	36	5	
	2	28	7	
	3	5	1	
	4	0	1	
Solitary Kidney		5.4%	0%	0.298
Stone opacity				0.352
	Opaque	87	19	
	Non opaque	5	0	
Previous SWL history		54.3%	47.4%	

**SWL** = Shock Wave Lithotripsy

**Table 4:** Operative and post-operative characteristics of patients with multiple renal stones according to absence/presence of lower pole stone.

	Lower calyceal stone present	No lower calyceal stone	P value
Number	92	19	
Type of f-URS			0.810
Flex-X2 (Fiberoptic)	72	15	
Dur-D (Digital)	20	4	
Mean operation time(minutes)	47.1±22.1	51.5±23.2	0.431
Mean fluoroscopy time(minutes)	2.6±1.4	2.3±1.8	0.436
Mean hospitalisation time(hours)	21.1±13	29.3±28.5	0.052
Post operative complications			0.616
Severe pain (grade 1)	14(15.2)	2(10.6%)	
Need for antibiotic change due to fever (grade 2)	4(4.4%)	1(5.3%)	
Gross bleeding (grade 1)	1(1.1%)	1(5.3%)	
JJ Catheter migration (grade 3a)	1 (1.1%)	0	
JJ Catheter migration (grade 3b)	1 (1.1%)	1 (5.3%)	
Peroperative success			0.856
Stone free status	80(87.0)	16(84.2%)	
Residual stone	12(13.0)	3(15.8%)	
Need for additional procedures	2(2.2%)	1(5.3%)	0.153
Stone analysis			0.977
Calcium oxalate monohydrate	31	6	
Calcium oxalate dihydrate	13	2	
Uric acid	2	0	
Cystine	6	1	
Struvite	2	0	
Mixt	11	2	

**JJ catheter** = Double-J catheter

## DISCUSSION

The treatment recommendation for kidney stone(s) in urolithiasis guidelines substantially depends on stone burden and location of the stone. However, many studies had demonstrated stone number's effect on the success and complications of the treatment modality, especially in SWL. We believe that number of stones can effect the decision of treatment modality and this variable should be added in urolithiasis guideline's recommendations. Thus, we investigate the efficiency of f-URS in multiple stones, where SWL is recommended as the first line treatment option, according to stone size.

The efficacy and safety of SWL on single kidney stone under 300mm^2^ is well described, however, success rate of the procedure declines as the number of stones increase. Cash et al. reported ≤50% stone free rate after SWL, in the treatment of multiple intrarenal stones (8). McAdams et al. performed SWL in 149 patients and 32 of them had multiple renal stones. The mean number of stones was 1.87 in successfully treated patients and 2.81 in those with treatment failure, in their study (p=0.065) (9). In addition, stone free rates decrease below 70% for single renal stone located in the lower pole (10). Thus, we believe SWL is not a suitable option for multiple renal stones, especially if one or more stone(s) are located in the lower pole.

Percutaneous nephrolithotomy is another treatment alternative for renal stone(s) under 300mm^2^, with excellent stone free rates up to 95% in one procedure. However, PNL is associated with increased complication rates when compared to SWL or f-URS (11). Renal parenchymal injury is ineluctable in PNL procedure and adjacent organ injuries mostly occur while performing access. In cases where multiple kidney stones are located in more than one location, one access may not be enough.

Previous studies had demostrated that multiple access was associated with a greater amount of blood loss, discomfort and renal function deterioration.

We achieved 92.7% and 86.4% stone free status in patients with solitary renal stone and multiple renal stones, respectively. Both Breda et al. and Huang et al. reported 100% success with f-URS in multiple renal stones under 20mm (12, 13). However, their study samples involved less number of patients when compared with our study and included 51 and 25 patients, respectively. Additionally, we performed only 3 (2.3%) second f-URS procedure in patients with multiple renal stones. In Huang's study, 9 patients (36%) required second f-URS procedure and 2 patients (8%) required third f-URS procedure. Similarly, Breda et al. performed 1.4 f-URS procedure while getting a 100% success rate. Additionally, focusing the stone during stone fragmentation is more difficult in hydronephrotic cases, due to increased mobility of the stone. We have encountered more hydronephrotic cases in group 2 and this might be another reason for lower stone free rates and longer operation time in this group.

Treatment of lower pole stones is challenging for urologists because of anatomic variabilities and technical conditions. Nonetheless, we did not shown any significant difference on success in patients with multiple stones, whether patients have lower pole stone(s) or not (p=0.856). Similar to our results, Jacquemet et al. compared 139 and 232 f-URS procedures for stones located in a single renal location other than the lower pole and at least one stone located in the lower pole, respectively. They did not found any impact of lower pole localization on f-URS success (14). Although preoperative characteristics were well matched between our patients with multiple stones, we compared only 92 patients and 19 patients, with and without lower pole stones, retrospectively. We believe studies with high patient volume will clarify this issue.

The mean operation time was longer in patients with multiple renal stones (47.8 min vs. 44.6 min) but the difference was not statistically significant (p=0.229). We believe that determining the location of the stones in different localizations and focusing with the laser is a time consuming process. Our operation time was found shorter compared to similar studies which evaluate operation time in multiple renal stones. The mean operation time was 65.1 min in Breda's study but they calculated the operation time from the time of cystoscope insertion to the stent placement, different from our study. Additionally, their study included patients with 20mm stone size (12). Huang et al. calculated the operation time similar to our study but they informed the mean operation time per patient (81.2 min) including second and third procedures, if necessary (13).

We found that the mean fluoroscopy screening time was significantly longer in patients with multiple renal stones (p=0.043). Previous reports discussed above did not mention about fluoroscopy screening time. However, we are aware that our fluoroscopy screening time was longer when compared to other f-URS studies in the literature. We believe that our tendency to frequently determine the location of the stone with retrograde pyelography and aid fluoroscopy screning to find the stone(s) resulted in longer fluoroscopy screening time.

Complication rates were 15.3% and 23.4% in patients with solitary renal stone and multiple renal stones, respectively but difference was not statistically significant. We did not face any Clavien 4 and Clavien 5 complications. Severe pain was the most common post operative complication in both groups. Urinary tract infection was seen only in the multiple renal stones group. We deemed that more manipulation in multiple renal stones was related with severe pain and infectious complications. Double-J stent migration was identified in five patients with a kidney – ureter – bladder radiogram postoperatively. The double-J stent was reinserted under local anesthesia in two patients (Clavien 3a) and under general anesthesia in three patients (Clavien 3b), respectively.

Although with sufficient number of patients, our study has some limitations. First of all, we are aware of the retrospective nature of our study. Secondly, we did not evaluate the need of analgesic requirements postoperatively and cost of our procedures. Also, comparison of the presence of lower pole stones were made between unevenly numbered groups. Additionally, our stone analysis datas are incomplete, which is another limitation of our study. Our Ministry of Health does not provide a free ‘Stone analysis’ for every patient, thereby, only 132 of our patients had stone analysis results. Finally, secondary procedures for residual stone fragments was not commonly applied to our patients, because most of our patients were symptom-free and did not want to undergo a secondary procedure.

## CONCLUSIONS

Our study has showed that f-URS achieved better stone free status in solitary renal stone than multiple renal stones under 300mm^2^. However, stone free status and complication rates of f-URS were acceptable in patients with multiple stones. To our knowledge, this is the first study which compares the effect of multiple stones or single stone on the outcomes of f-URS. However, our findings must be supported by further prospective, randomized studies with large patient volume.

## ABBREVIATIONS

SWL: = Shock Wave Lithotripsy
PNL: = Percutaneous Nephrolithotomy
F-URS: = Flexible ureterorenoscopy
IVU: = Intravenous urography
CT: = Computed tomography
BMI: = Body mass index
Min: = Minutes
